# Mapping of a novel locus associated with autosomal recessive congenital cataract to chromosome 8p

**Published:** 2010-12-30

**Authors:** Namerah Sabir, S. Amer Riazuddin, Haiba Kaul, Farheena Iqbal, Idrees A. Nasir, Ahmad U. Zafar, Zaheeruddin A. Qazi, Nadeem H. Butt, Shaheen N. Khan, Tayyab Husnain, J. Fielding Hejtmancik, Sheikh Riazuddin

**Affiliations:** 1 National Centre of Excellence in Molecular Biology, University of the Punjab, Lahore, Pakistan; 2 The Wilmer Eye Institute, Johns Hopkins University School of Medicine, Baltimore, MD; 3 Layton Rahmatulla Benevolent Trust Hospital, Lahore, Pakistan; 4 Allama Iqbal Medical College, University of Health Sciences, Lahore, Pakistan; 5 Ophthalmic Genetics and Visual Function Branch, National Eye Institute, National Institutes of Health, Bethesda, MD

## Abstract

**Purpose:**

To identify the disease locus for autosomal recessive congenital cataracts in a consanguineous Pakistani family.

**Methods:**

All affected individuals underwent a detailed ophthalmologic examination. Blood samples were collected and genomic DNA was extracted. A genome-wide scan was completed with fluorescently-labeled microsatellite markers on genomic DNA from affected and unaffected family members. Logarithms of odds (LOD) scores were calculated under a fully penetrant autosomal recessive model of inheritance.

**Results:**

Ophthalmic examination suggested that affected individuals have bilateral cataracts. Linkage analysis localized the critical interval to chromosome 8p with LOD scores of 3.19, and 3.08 at θ=0, obtained with markers D8S549 and D8S550, respectively. Haplotype analyses refined the critical interval to 37.92 cM (16.28 Mb) region, flanked by markers, D8S277 proximally and D8S1734 distally.

**Conclusions:**

Here, we report a new locus for autosomal recessive congenital cataract mapped to chromosome 8p in a consanguineous Pakistani family.

## Introduction

Congenital cataracts are the principal cause of visual impairment in children worldwide and are responsible for about one third of cases of blindness in infants [1,2]. The prevalence of non-syndromic cataract is estimated at 1–6 cases per 10,000 live births in industrialized countries whereas these numbers are estimated to be much higher in developing conuntaries [3-6]. Cataracts can lead to permanent blindness by interfering with the sharp focus of light on the retina, especially during the early developmental periods. Cataracts are classified according to their morphology, and/or the location of opacity in the lens [7].

Approximately one-third of congenital cataract cases are familial [8], and to-date fourteen loci have been associated with autosomal recessive cataracts with seven of these have causally associated with autosomal dominant cataracts [9-22]. Of these loci, pathogenic mutations in nine genes, eph-receptor type-A2 (*EPHA2*), connexin50 (*GJA8*), glucosaminyl (N-acetyl) transferase 2 (*GCNT2*), heat-shock transcription factor 4 (*HSF4*), lens intrinsic membrane protein (*LIM2*), beaded filament structural protein 1 (*BFSP1*), alphaA-crystallin (*CRYαA*), betaB1-crystallin (*CRYβB1*), and betaB3-crystallin (*CRYβB3*) have been identified [9,11,13,16,18-22].

Here, we report a consanguineous Pakistani family with multiple affected individuals. Linkage analysis localized the critical interval to chromosome 8p with a significant Lod scores and haplotype analyses refined the critical interval to a 37.92 cM (16.28 Mb), flanked by markers D8S277 proximally and D8S1734 distally.

## Methods

### Clinical ascertainment

A total of 125 consanguineous Pakistani families with nonsyndromic cataracts were recruited to participate in a collaborative study between the National Centre of Excellence in Molecular Biology, Lahore, Pakistan, and the National Eye Institute, Bethesda, MD, to identify novel loci associated with congenital cataracts. Institutional Review Board (IRB) approval was obtained from the both Institutes and all participating subjects gave informed consent consistent with the tenets of the Declaration of Helsinki. A detailed medical history was obtained by interviewing family members. Ophthalmic examinations were conducted with slit-lamp microscopy. Approximately 10 ml of blood samples were drawn from affected and unaffected members of the family and stored in 50 ml Sterilin® falcon tubes (BD Biosciences, San Jose, CA) containing 400 μl of 0.5 M EDTA. Blood samples were kept at −20 °C for long- term storage.

### DNA extraction

DNA was extracted by a nonorganic method as described previously [9,14]. Briefly, aliquots of 10 ml blood samples were mixed with 35 ml of TE buffer (10 mM Tris-HCl, 2 mM EDTA, pH 8.0) and the TE-blood mixture was centrifuged at 1,800× g for 20 min. The supernatant was discarded and the pellet was re-suspended in 35 ml of TE buffer and centrifuged at 1,800× g for 20 min. The TE washing was repeated for 2–3 times and the washed pellet was re-suspended in 2 ml of TE. 6.25 ml of protein digestion cocktail (50 μl [10 mg/ml] of proteinase K, 6 ml TNE buffer [10 mM Tris HCl, 2 mM EDTA, 400 mM NaCl] and 200 μl of 10% sodium dodecyl sulfate) was added to the re-suspended pellets and incubated overnight in a shaker (250 rpm) at 37 °C. The digested proteins were precipitated by adding 1 ml of 5 M NaCl, followed by vigorous shaking and chilling on ice for 15 min. The precipitated proteins were pelleted by centrifugation at 1,800× g for 20 min and removed. The supernatant was mixed with equal volumes of phenol/chloroform/isoamyl alcohol (25:24:1) and the aqueous layer containing the genomic DNA was carefully collected. The DNA was precipitated with isopropanol and pelleted by centrifugation at 2,400× g) for 15 min. The DNA pellets were washed with 70% ethanol and dissolved in TE buffer. The DNA concentration was determined with a SmartSpec plus Bio-Rad Spectrophotometer (Bio-Rad, Hercules, CA).

### Genotype analysis

A genome-wide scan was performed with 382 highly polymorphic fluorescent markers from the ABI PRISM Linkage Mapping Set MD-10 (Applied Biosystems, Foster City, CA) having an average spacing of 10 cM. Multiplex polymerase chain reaction (PCR) was completed in a GeneAmp PCR System 9700 thermocycler (Applied Biosystems). Briefly, each reaction was performed in a 5 μl mixture containing 40 ng genomic DNA, various combinations of 10 mM dye-labeled primer pairs, 0.5 ml 10× GeneAmp PCR Buffer (Applied Biosystems), 1 mM dNTP mix, 2.5 mM MgCl_2_, and 0.2 U *Taq* DNA polymerase (Applied Biosystems). Initial denaturation was performed for 5 min at 95 °C, followed by 10 cycles of 15 s at 94 °C, 15 s at 55 °C, and 30 s at 72 °C and then 20 cycles of 15 s at 89 °C, 15 s at 55 °C, and 30 s at 72 °C. The final extension was performed for 10 min at 72 °C. PCR products from each DNA sample were pooled and mixed with a loading cocktail containing HD-400 size standards (Applied Biosystems). The resulting PCR products were separated in an ABI 3100 DNA Analyzer (Applied Biosystems) and genotypes were assigned with GeneMapper software (Applied Biosystems).

### Linkage analysis

Two-point linkage analyses were performed using the FASTLINK version of MLINK from the LINKAGE Program Package, whereas the Maximum LOD scores were calculated with ILINK from the LINKAGE Program Package [23,24]. Autosomal recessive cataract was analyzed as a fully penetrant trait with an affected allele frequency of 0.001. The marker order and distances between the markers were obtained from the Marshfield database and the National Center for Biotechnology Information (NCBI) chromosome 8 sequence maps. For the initial genome scan, equal allele frequencies were assumed, while for fine mapping allele frequencies were estimated from 96 unrelated and unaffected individuals from the Punjab province of Pakistan.

## Results

**Figure 1:**
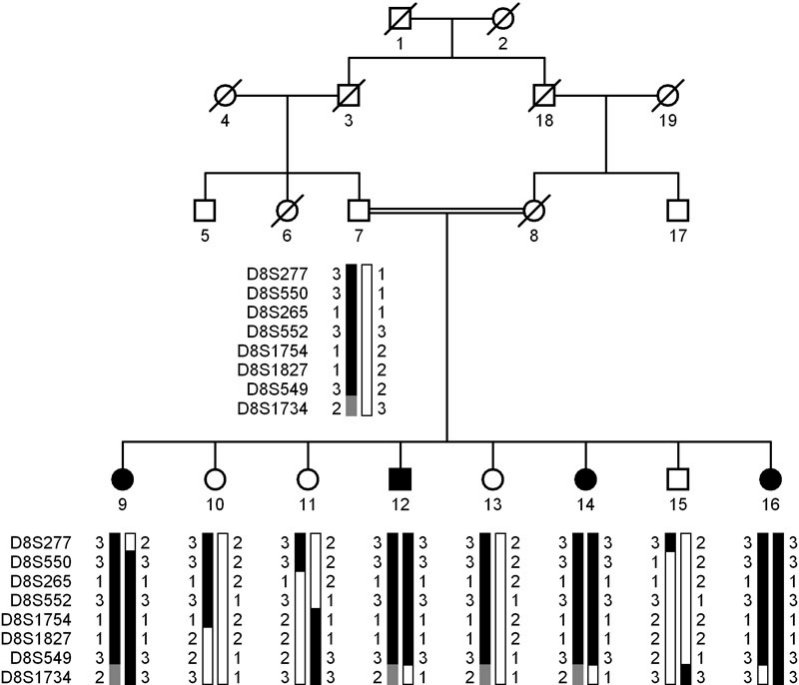
Pedigree drawing and haplotypes of chromosome 8p markers of family PKCC146. Squares are males, circles are females, and filled symbols are affected individuals; the double line between individuals indicates consanguinity and the diagonal line through a symbol is a deceased family member. The haplotypes of 8 adjacent microsatellite markers are shown with alleles forming the risk haplotype shaded black, alleles co-segregating with cataracts but not showing homozygosity are shaded gray and alleles not co-segregating with cataracts are shown in white.

**Figure 2:**
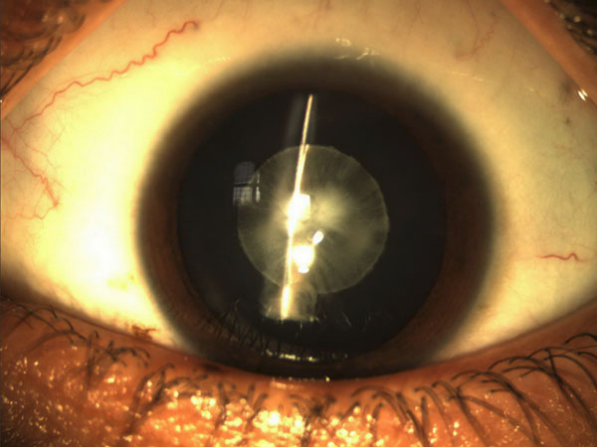
Slit lamp photograph of the affected individual 16 of family PKCC146 show laminar and nuclear cataracts that developed in early infancy.

A large consanguineous family, PKCC144, consisting of four affected and four unaffected individuals was recruited from the Punjab province of Pakistan (Figure 1). The medical records of available to us for PKCC146 revealed that all affected individuals developed cataracts in their infancy. Medical records of previously conducted ophthalmic examinations with slit lamp biomicroscopy were suggestive of bilateral cataracts; we were able to further characterize the phenotype to laminar and nuclear cataracts in affected individual 15 (Figure 2). None of the affected individuals presented any extra-ocular anomalies.

Initially, linkage to all reported loci associated with autosomal recessive cataract loci were excluded by haplotype analyses with closely spaced fluorescently-labeled microsatellite markers (data not shown). Subsequently, a genome-wide scan was completed and during the genome-wide scan maximum two-point LOD scores of 3.19, and 3.08 at θ=0 were obtained with markers D8S550 and D8S549, respectively (Table 1). Additional STR markers from the Marshfield database were designed to analyze the critical interval, which further provided additional evidence of linkage to chromosome 8p with two-point LOD scores of 2.09, 2.01, 2.90, and 3.15 at θ=0 with markers D8S265, D8S552, DS1754, and D8S1827, respectively (Table 1).

**Table 1:** Two-point LOD scores of chromosome 8p markers.

**Marker**	**cM**	**Mb**	**0**	**0.01**	**0.05**	**0.09**	**0.10**	**0.20**	**0.30**	**Z_max_**	**θ_max_**
D8S277*	8.34	6.51	-∞	−2.59	−0.73	−0.2	−0.12	0.12	0.20	0.20	0.30
D8S550*	21.33	10.88	3.19	3.11	2.84	2.57	2.51	1.95	1.25	3.19	0.00
D8S265	21.87	11.27	2.09	2.04	1.86	1.68	1.63	1.17	0.72	2.09	0.00
D8S552	26.43	12.74	2.01	1.96	1.78	1.60	1.55	1.09	0.68	2.01	0.00
D8S1754	27.40	12.98	2.90	2.82	2.56	2.28	2.24	1.66	0.98	2.90	0.00
D8S1827	30.49	14.81	3.15	3.07	2.81	2.53	2.48	1.92	1.21	3.15	0.00
D8S549*	31.73	15.64	3.08	3.00	2.75	2.45	2.41	1.85	1.16	3.08	0.00
D8S1734*	46.26	22.79	-∞	−5.25	−2.61	−1.74	−1.60	−0.02	0.05	0.05	0.30

The asterisk indicates markers used in the genome-wide scan.

Visual inspection of the haplotypes supported the results of linkage analysis (Figure 1). There is a recombination in affected individual 9 at D8S277 that defines the proximal boundary, whereas recombination events in affected individuals 12, 14, and 16 at D8S1734 define the distal boundary (Figure 1). This places the critical interval in a 37.92 cM (16.28 Mb) interval flanked by D8S277 proximally and D8S1734 distally. Alleles of markers D8S550, D8S265, D8S552, D8S1754, D8S1827, and D8S549 are homozygous in all affected individuals.

## Discussion

Here, we report a new locus for autosomal recessive congenital cataracts localized to chromosome 8p in a consanguineous Pakistani family. The medical records available to us suggest a congenial onset, whereas the slit lamp examination was suggestive of bilateral cataracts. The genome-wide scan localized the critical interval to chromosome 8p, which was further supported by haplotype analyses and places the critical interval in a 37.92 cM (16.28 Mb) region, flanked by markers D8S277 proximally and D8S1734, distally.

While the maximum Lod score of 3.19 is only slightly higher than the traditional limiting value of 3.0, it represents the maximum value obtainable with this family with real allele frequencies. In addition, the lack of evidence for linkage during the genome-wide scan, except with markers D8S549 and D8S550 provides additional support for localization of the cataract locus to chromosome 8p. This is the first report associating chromosome 8p with autosomal recessive congenital cataracts and illustrates the heterogeneity of the disease.

The critical interval is a gene rich region that harbors more than 100 genes according to the UCSC database. We have prioritized all annotated genes based on their known function and available expression data in the eye, and currently are sequencing each candidate in one affected and one unaffected individual. Identification of the pathogenic mutations that lead to congenital cataracts will increase our understanding of lens biology at a molecular level and will help in development of better treatments and therapeutics.
